# Identifying the Neural Correlates of Resting State Affect Processing Dynamics

**DOI:** 10.3389/fnimg.2022.825105

**Published:** 2022-04-21

**Authors:** Kevin P. Fialkowski, Keith A. Bush

**Affiliations:** Brain Imaging Research Center, University of Arkansas for Medical Sciences, Little Rock, AR, United States

**Keywords:** affect, emotion, dynamics, resting state, fMRI, decoding, MVPA

## Abstract

There exists growing interest in understanding the dynamics of resting state functional magnetic resonance imaging (rs-fMRI) to establish mechanistic links between individual patterns of spontaneous neural activation and corresponding behavioral measures in both normative and clinical populations. Here we propose and validate a novel approach in which whole-brain rs-fMRI data are mapped to a specific low-dimensional representation—affective valence and arousal processing—prior to dynamic analysis. This mapping process constrains the state space such that both independent validation and visualization of the system's dynamics become tractable. To test this approach, we constructed neural decoding models of affective valence and arousal processing from brain states induced by International Affective Picture Set image stimuli during task-related fMRI in (*n* = 97) healthy control subjects. We applied these models to decode moment-to-moment affect processing in out-of-sample subjects' rs-fMRI data and computed first and second temporal derivatives of the resultant valence and arousal time-series. Finally, we fit a second set of neural decoding models to these derivatives, which function as neurally constrained ordinary differential equations (ODE) underlying affect processing dynamics. To validate these decodings, we simulated affect processing by numerical integration of the true temporal sequence of neurally decoded derivatives for each subject and demonstrated that these decodings generate significantly less (*p* < 0.05) group-level simulation error than integration based upon decoded derivatives sampled uniformly randomly from the true temporal sequence. Indeed, simulations of valence and arousal processing were significant for up to four steps of closed-loop simulation (Δt = 2.0 s) for both valence and arousal, respectively. Moreover, neural encoding representations of the ODE decodings include significant clusters of activation within brain regions associated with affective reactivity and regulation. Our work has methodological implications for efforts to identify unique and actionable biomarkers of possible future or current psychopathology, particularly those related to mood and emotional instability.

## Introduction

Emotional experiences unfold over time (Zelazo and Cunningham, 2007; Cunningham et al., 2013). Thus, knowledge of normative emotion processing dynamics may be particularly important for informing our understanding of mental well-being. Indeed, emotion dysregulation, which is implicated in maladaptive trajectories of emotion processing, has been linked to depression, borderline personality disorder, substance-use disorders, eating disorders, somatoform disorders, and other psychopathology (Berking and Wupperman, 2012). At present, however, we know little of the neural mechanisms through which emotion processing temporally evolves.

Along these lines, the elucidation of affect processing within the untasked brain has been an area of recent inquiry. Mind wandering is the default mode of human brain activity (Raichle et al., 2001), comprising as much as 47% of all wakeful cognitive states (Killingsworth and Gilbert, 2010). Interestingly, observations have been made in studies of mind wandering that show diverse, often negative, self-reported affective experiences (Killingsworth and Gilbert, 2010; Kragel et al., 2016). Evidence from a large-scale (*n* = 2,500) ecological momentary assessment study suggests that mind wandering is a cause, rather than an effect, of negative affect (Killingsworth and Gilbert, 2010). However, a brain-based mechanistic explanation for this phenomenon remains poorly described. Advancements in methods for analyzing resting state brain data, therefore, may hold promise in expanding our overall understanding of affect processing dynamics.

Neural signatures of mind wandering are typically captured via resting state functional magnetic resonance imaging (rs-fMRI) while the subject remains inactive, visually fixating on crosshairs, and under instructions to let the mind freely wander. Standard analysis methods currently utilize functional connectivity (FC) to characterize the temporal correlations between neural activations measured from disparate spatial regions throughout the brain. Using the FC approach, researchers have demonstrated that the functional organization of the resting brain broadly recapitulates task-related cognitive processing (Gordon et al., 2017), thereby allowing patterns of rs-fMRI brain activity to predict cognitive and behavioral states as well as to infer mechanisms of cognitive development (Dosenbach et al., 2010; Gu et al., 2015) and deficit states (Greicius et al., 2004, 2007; Cisler et al., 2013). It is this broad informing potential of rs-fMRI that prompted its inclusion in population-scale normative adult (Smith et al., 2013) and developmental (Brown et al., 2015; Casey et al., 2018) imaging studies.

However, rs-fMRI temporal and spatial pattern analyses have inherent theoretical, conceptual, and interpretive limitations to their ultimate potential for improving our understanding of the mechanisms of mind wandering. First (in what we term the *dynamics problem*), existing rs-fMRI FC analyses largely rely on static topologies of functional brain organization (Bullmore and Sporns, 2009) in which steady-state network structures (e.g., default mode network) emerge from correlations between coarse-grained anatomical parcellations (Glasser et al., 2016) of fMRI BOLD signal (Buxton et al., 2004). Second (in what we term the *identity problem*), fluctuating rs-fMRI patterns of neural activation lack defined cognitive process identities that directly relate individual and group variation to the extant task-related functional neuroanatomical literature, thus diminishing their potential value for informing our understanding of cognitive processes (and their interactions) within the untasked brain.

Highly innovative work has recently approached the dynamics problem by decomposing the steady-state functional organization of the human brain into temporally finer-grained, moment-to-moment dynamics of resting state FC reorganization (Liu and Duyn, 2013; Calhoun et al., 2014; Gonzalez-Castillo et al., 2015), which appears strikingly similar to FC reorganization that has been observed when the brain alternates between tasks or between task and rest (Gonzalez-Castillo et al., 2015). This approach suggests that modes of resting state FC are governed, macroscopically, by a stochastic process (Parzen, 2015) such as a Markov chain (Privault, 2013; Kragel et al., 2021).

Recent work has approached the identity problem by demonstrating that cognitive-like identities spontaneously and dynamically emerge within rs-fMRI data (Gonzalez-Castillo et al., 2019). Further, advances in multivariate neural decoding (Mitchell et al., 2008) have provided insight into the specific cognitive process identities that are corollary to free-forming rs-fMRI neural activity. Both emotion processing (Kragel et al., 2016) and affect processing (Bush et al., 2018c) identities have been decoded from rs-fMRI data and independently validated, either by self-reported ecological affective experience (Kragel et al., 2016) or by concurrently recorded psychophysiology (Bush et al., 2018c).

Building on these separate findings, we propose that innovative work must investigate methodologies that merge the analysis of resting state dynamics with specific cognitive identities in order to provide insight into the processing and regulatory mechanisms involved. One candidate approach that has embraced this exploratory framework was recently presented by Kragel et al. (2021). In this approach, a low-dimensional set of emotion identities (anger, contentment, fear, happiness, sadness, surprise, and neutral) were neurally decoded from each timepoint of the subjects' rs-fMRI data and then subjected to dynamic analysis according to a Markov process, yielding the underlying probabilities of transitioning between each emotional identity. While this approach shed light on the critical link between emotion transition dynamics and psychopathology, it was limited by its inability to elucidate the neural mechanisms underlying the transition function.

In seeking a uniquely innovative approach to rs-fMRI dynamic analysis, this study explored the continuous dimensional space of affective valence and arousal rather than the discrete space of basic emotions. We applied previously reported, psychophysiologically validated neural decoding models of affective valence and arousal processing to characterize the affective experiences evolving within the untasked brain during rs-fMRI acquisition (Bush et al., 2018c). Next, we calculated the first and second temporal derivatives of the respective valence and arousal time-series and subsequently fit additional decoding models to these data. These decodings represent neurally constrained ordinary differential equations (ODE) underlying affect processing dynamics. The goal of this work was to test the validity of these ODEs in describing resting state affect processing dynamics and elucidate the functional neural correlates driving this dynamical system.

## Methods

### Analysis Overview

The data for this analysis were drawn from two existing studies, the Intrinsic Neuromodulation of Core Affect (INCA) experiment and the Cognitive Control Theoretic Mechanisms of Real-time fMRI-Guided Neuromodulation (CTM) experiment (National Science Foundation, BCS-1735820). Both studies shared identical MRI acquisitions up to and including the resting state task data analyzed herein. In brief, the INCA and CTM studies were conducted across two sessions, each occurring on separate days. Within Session 1, participants were consented, screened for clinically relevant exclusionary criteria, and behaviorally assessed. Within Session 2, participants underwent a T1-weighted structural acquisition, two affect induction fMRI task acquisitions, and a resting state fMRI task acquisition, as well as two additional real-time fMRI task acquisitions, which are not part of this analysis. We have reported the details of this combined dataset and our analysis methods previously (Bush et al., 2020). For clarity of this current analysis, we explicitly report salient details of our participant sample as well as our neuroimaging acquisition and data processing pipelines.

### Participants

The combined participant sample (*n* = 97) of the CTM and INCA studies was the maximum number of healthy control subjects available with the affect induction and resting state fMRI acquisitions necessary to conduct the analysis. From this sample, we excluded three participants (one INCA participant was incorrectly included despite meeting exclusion criteria and two CTM participants failed to complete the resting state scan due to early exit from the scanner). Thus, the final combined dataset of these studies (*n* =94; n_CTM_ = 75 and n_INCA_ = 19) was comprised of participants exhibiting the following demographic characteristics: age [mean(s.d.)]: 36.6(13.8), range 18–64; sex: 61(65%) female, race/ethnicity: 80(85.1%) self-reporting as White or Caucasian, 11(11.7%) as Black or African-American, 1(1.1%) as Asian, and 2 (2.1%) reporting as more than one race; education [mean(s.d.)]: 16.7(2.6) years, range 12–23; WAIS-IV IQ [mean(s.d.)]: 105.8(14.0), range 74–145. All participants were right-handed native-born United States citizens, were medically healthy, and exhibited no current Axis I psychopathology as assessed by the SCID-IV clinical interview (American Psychiatric Association, 1994). Participants reported no current use of psychotropic medication and produced a negative urine screen for drugs of abuse (cocaine, amphetamines, methamphetamines, marijuana, opiates, and benzodiazepines) immediately prior to the MRI scan. Further, CTM participants produced a negative urine screen prior to SCID-IV clinical interview. When necessary, participants' vision was corrected to 20/20 using an MRI compatible lens system (MediGoggles™, Oxfordshire, United Kingdom). Participants endorsing color blindness were excluded.

### Ethics Statement

All participants provided written informed consent after receiving written and verbal descriptions of the study procedures, risks, and benefits. All study procedures and data analysis were performed with approval and oversight of the Institutional Review Board at the University of Arkansas for Medical Sciences (UAMS) in accordance with the Declaration of Helsinki and relevant institutional guidelines and policies.

### Task Design

The System Identification fMRI task acquisition consisted of two 9.4 min scans during which the participant was presented with 120 images that were computationally sampled from the International Affective Picture System (Lang et al., 2008) (IAPS) in order to induce the maximum span of arousal-valence experiences. Details of these task stimuli have been extensively reported (Bush et al., 2018a,c; Wilson et al., 2020). Image stimuli were presented according to two pseudo randomly sequenced trial types: implicit affect induction (90 images) and cued-recall/re-experiencing (Bush et al., 2020) (30 images). This analysis was informed by fMRI data acquired during implicit affect induction trials, which are characterized by a 2 s presentation of the image stimulus followed by an intertrial interval uniformly randomly sampled from the range 2–6 s during which a white fixation cross was presented on a black background. The Resting State task acquisition consisted of one 7.5 min resting state fMRI scan in which a white fixation cross was displayed on a black background throughout. During pre-acquisition training for this task, participants were instructed to “let your mind wander, not focusing on any specific thought” and to “try to keep your head still and your eyes open” but to “blink naturally.”

### MR Image Acquisition and Preprocessing

All imaging data were acquired using the same Philips 3T Achieva X-series MRI scanner (Philips Healthcare, Eindhoven, The Netherlands) with a 32-channel head coil. Anatomic images were acquired using an MPRAGE sequence (matrix = 256 × 256, 220 sagittal slices, TR/TE/FA = 8.0844 ms/3.7010 ms/8°, final resolution = 0.94 × 0.94 × 1 mm^3^). Functional images were acquired using the following EPI sequence parameters: TR/TE/FA = 2,000 ms/30 ms/90°, FOV = 240 × 240 mm, matrix = 80 × 80, 37 oblique slices, ascending sequential slice acquisition, slice thickness = 2.5 mm with 0.5 mm gap, final resolution 3.0 x 3.0 x 3.0 mm^3^. All MRI preprocessing and neuroimage manipulations were performed using AFNI (Cox, 1996) (Version AFNI_19.1.04) unless otherwise noted. Anatomical data were processed via the following sequence of steps: skull stripping, spatial normalization to the MNI152 brain atlas, and segmentation (via FSL; Jenkinson et al., 2012) into white matter (WM), gray matter (GM), and cerebrospinal fluid (CSF). A group-level GM mask was constructed from individual participant GM segmentations that included voxels identified as GM for ≥ 50% of individuals. Functional neuroimages were processed according to the following sequence of steps: despiking, slice-time correction, deobliquing, motion correction, transformation to the spatially normalized anatomic image, regression of the mean time courses and temporal derivatives of the WM and CSF masks as well as a 24-parameter motion model (Power et al., 2012, 2015), spatial smoothing (8 mm FWHM Gaussian kernel), and scaling to percent signal change. The global mean signal was subtracted from resting state functional images prior to smoothing and scaling.

### Computational Modeling

#### Constructing and Validating Decoding Models of Affect Processing

Following methodology that has been extensively reported (Bush et al., 2018b,c; Wilson et al., 2020), within-subject neural decoding models of affect processing were constructed for each participant as follows. For each implicit affect induction trial, affect processing brain states were extracted from the fMRI BOLD signal via the beta-series method (Rissman et al., 2004) using the AFNI 3dDeconvolve function's individual modulation configuration followed by regression via the AFNI 3dLSS function. In essence, the beta-series method permits the generation of a general linear model consisting of individual hemodynamic response function regressors for each task-related trial. This preserves individual trial variance when compared to conventional methods that average the beta coefficients fit to each trial. The extracted brain states were then paired with the IAPS normative scores associated with the individual stimuli and binarized to affective class labels {+1,-1}, respectively, for the valence and arousal dimensions of affect, according to the middle Likert score (5 on a 9-point scale). These brain states and class labels were then used as feature-label pairs to train linear support vector machine classifiers of affect processing using Matlab's fitcsvm function with default hyperparameters (i.e., solution via the Iterative Single Data Algorithm (Kecman et al., 2005), penalty = 1 for all misclassifications, and solution tolerance = 0.001). Each within-subject model's classification accuracy was measured according to a leave-one-out cross validation scheme in which, upon removal of each hold-out feature-label pair, the remaining feature-label pairs were randomly sampled (30 times) to insure that the null classification probability remained 50%, respectively, for both valence and arousal (see prior work; Bush et al., 2018c).

#### Decoding of Resting State Affect Processing

**Figure 1:**
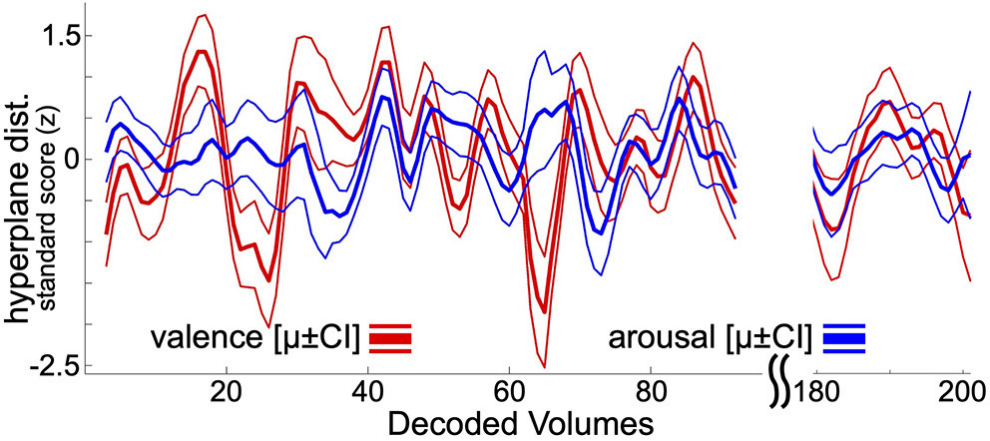
Resting state neural decodings of affect processing. This figure depicts an example neural decoding of affect processing for a participant involved in this study. Bold lines depict out-of-sample ensemble-average predicted hyperplane distances of the presented participant's affect processing during resting state fMRI acquisition (225 volumes total). Thin lines represent the 95% confidence intervals of the distribution of mean. The plot excludes decodings from the first 5 and final 10 volumes of resting-state acquisition, which are discarded during the decoding process (see Methods). For visualization purposes, the plot also excludes decodings for resting state fMRI volumes 96 through 180.

Following previously reported and psychophysiologically validated methodology (Bush et al., 2018c), within-subject neural decoding models of affect processing were applied to decode the moment-to-moment affect processing taking place during resting state task fMRI acquisition. The focus here on resting state affect processing dynamics, rather than task-related affect processing dynamics, controls for potential ruleset maintenance and goal representation cognitive processes that would be entrained by task-related fMRI analysis. At present there is not a clean method for disambiguating lower-level affect processing dynamics from these higher-level cognitions. To summarize this approach, each hold-out participant's resting state acquisition is assumed to contain theoretical “self-task” stimuli (timepoints uniformly randomly sampled from the range of EPI volume acquisition times). These self-task stimuli are repeatedly sampled, transformed to brain states via the beta-series method, and decoded according to the ensemble average hyperplane distance (Dietterich, 2000) predicted by the linear SVM classifiers fit to the set of training participants (i.e., all other participants in the dataset). Repeatedly decoding these random self-task stimuli and grouping the decodings by EPI volume acquisition time yields resting state decoding estimates that converge to a stable temporal distribution of affect processing, respectively, for valence and arousal (see Figure 1). Each participant's resting state acquisition was assumed to contain 100 self-task stimuli which were randomly sampled 30 times to construct the decoding estimate. Significant outliers were replaced with undefined (i.e., NaN) values considering a nominal probability threshold of 0.05 and median/median absolute deviation (MAD) statistics using published methodology (Cox, 2002).

#### Estimating and Decoding Affect Processing Dynamics

Time-derivatives of the mean decoded affective arousal and valence were estimated according to a center divided difference method, which is known to have superior error profiles to single differencing methods (Thomas, 1995). Thus, numerical approximations of the first derivative and second derivative are provided by Equations 1 and 2 where x denotes the decoded affect property, xϵ{v,a}, such that:


(1) $$\begin{array}{r} \frac{dx}{dt}=\frac{x_{t+1}-x_{t-1}}{2},and \end{array}$$

(2) $$\begin{array}{r} \frac{d^{2}x}{dt^{2}}=\frac{{\frac{dx}{dt}}_{t+1}-{\frac{dx}{dt}}_{t-1}}{2}. \end{array}$$

The time-derivatives were calculated from the outlier corrected valence and arousal predictions. Within-subject linear SVM neural decoding models of these derivatives were then constructed, using identical LOOCV validation methodology as was performed for the affect processing decoding models. Here, however, the decodings are linear Matlab SVM regression models trained directly on the derivatives (via the fitrsvm function with default hyperparameters). Only non-NaN target data were utilized for model fitting, but no additional constraints were placed on the distribution of values used for training the decoding models.

#### Simulating Resting State Affect Processing

Numerical simulation of resting state affect processing was performed according to Equations (3, 4). For each volume of resting state fMRI BOLD data, the simulation state, s, was initialized according to the decoded valence and arousal properties of the brain state such that s = [v,a]. The first and second time-derivatives of this volume were also decoded and numerical integration proceeded according to the forward differencing method (Thomas, 1995) such that:


(3) $$\begin{array}{r} x_{t+1}=x_{t}+\frac{dx}{dt},and \end{array}$$

(4) $$\begin{array}{r} {\frac{dx}{dt}}_{t+1}={\frac{dx}{dt}}_{t}+\frac{d^{2}x}{dt^{2}}. \end{array}$$

Additional numerical integration steps proceeded via closed-loop (i.e., the current x and dx/dt were generated via numerical integration rather than substituting the decodings for those quantities at the current time-step). The simulation was conducted over all sequences for which numerical values of state and dynamics were present (i.e. not NaN) with no additional constraints.

#### Transforming Decoding Models to Neuroanatomical Encodings

To visualize the neural correlates of affect processing dynamics, a previously reported encoding transformation of our decoding models (Bush et al., 2018a) was used. The Haufe-transform (Haufe et al., 2014) was applied to each participant's decoding hyperplane. A map of group-level mean encoding values was then assembled for each gray matter voxel. Separately, 500 mean encoding permutations were generated by applying the Haufe-transform to the classification hyperplanes fit to each participant's true beta-series and randomly permuted sets of the true affective labels. Those voxels exhibiting extreme group-level mean encoding values in comparison to the observed group-level mean permutation encoding values (2-sided test, *p* < 0.05) were kept for visualization. This encoding process was performed separately for each dimension of affect processing (valence and arousal) and both the first and second derivatives as well as the base brain states (see Supplementary Figure 1). Only non-NaN target data were utilized for permutation model fittings.

## Results

### Decoding Models of Resting State Affect Processing Dynamics

**Figure 2:**
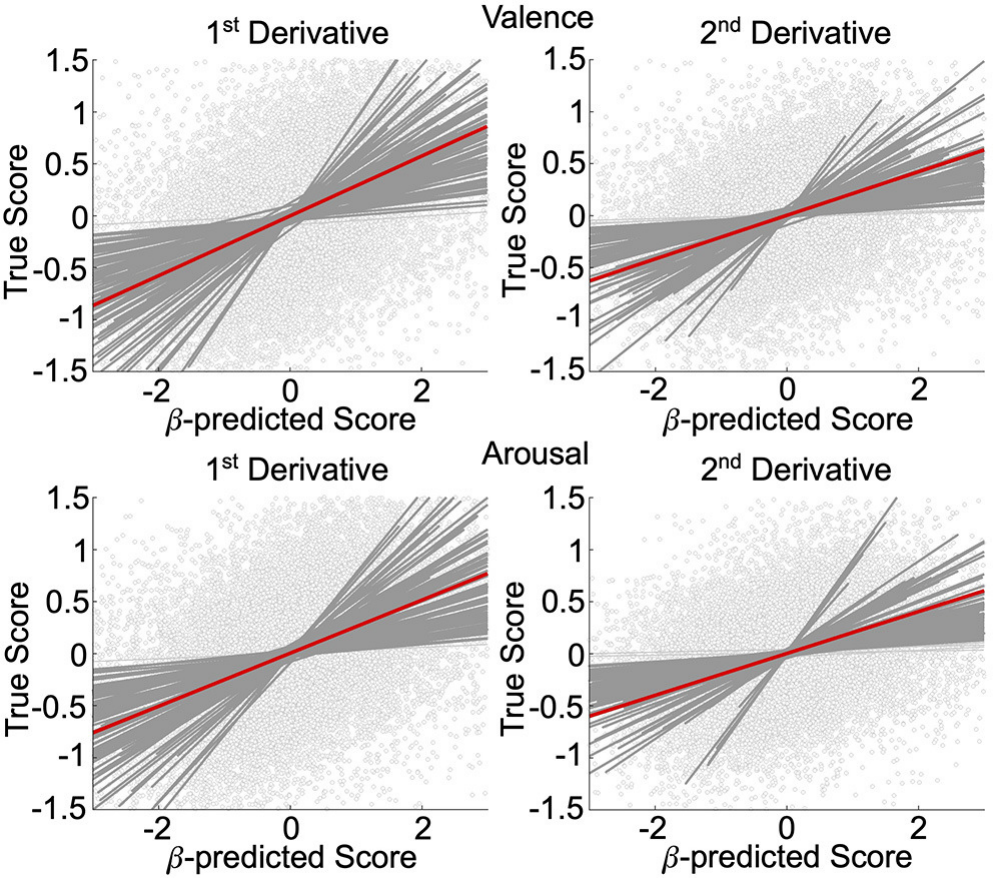
Affective dynamics decoding model validation. GLMM of the first and second temporal derivative within-subject rs-fMRI valence and arousal predictions made from rs-fMRI patterns of neural activation. Circle markers represent individual predictions. Red lines depict group-level prediction effects. Dark gray lines indicate significant subjects' effects. Light gray lines indicate nonsignificant subjects' effects.

We validated, using general linear mixed-effects models (GLMM), the neural decoding models of rs-fMRI temporal derivatives, depicted in Figure 2. Data is representative of *n* = 92 subjects whose rs-fMRI data survived motion thresholding. For all models, the measure-of-interest was the true derivative (numerically computed from the decoded rs-fMRI data), and the main fixed effect was the neurally decoded derivative. Random slope and intercept effects were modeled subject-wise. We demonstrated significant group-level evidence for neural decoding of affect processing dynamics. Specifically, for the first temporal derivative models, true normative valence scores were significantly predicted (fixed effect: β = 0.29, 95% CI [0.25, 0.33], *p* < 0.001, *t*-test, h_0_: β = 0) with effect size $R_{adj}^{2}$ = 0.24, and true normative arousal scores were significantly predicted (fixed effect: β = 0.26, 95% CI [0.22, 0.29], *p* < 0.001, *t*-test, h_0_: β = 0) with effect size $R_{adj}^{2}$ = 0.23. There were *n* = 90 (97.8%) and *n* = 91 (98.9%) subjects respectively that demonstrated within-subject significant effects (*p* < 0.05, *t*-test, h_0_: β = 0). For the second temporal derivative models, true normative valence scores were significantly predicted (fixed effect: β = 0.21, 95% CI [0.18, 0.24], *p* < 0.001, *t*-test, h_0_: β = 0) with effect size $R_{adj}^{2}$ = 0.16, and true normative arousal scores were also significantly predicted (fixed effect: β = 0.20, 95% CI [0.17, 0.24], *p* < 0.001, *t*-test, h_0_: β = 0) with effect size $R_{adj}^{2}$ = 0.16. There were *n* = 88 (95.7%) and *n* = 87 (94.6%) subjects, respectively, that demonstrated within-subject significant effects (*p* < 0.05, *t*-test, h_0_: β = 0).

### Simulation of Resting State Affect Processing Dynamics

**Figure 3:**
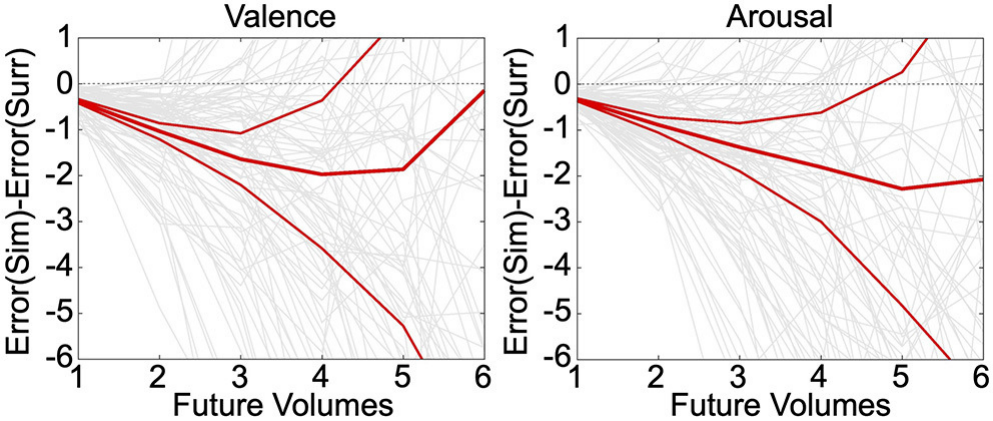
Simulation of affect processing trajectories. Neurally decoded first and second temporal derivatives of valence and arousal were used to simulate processing trajectories according to closed-loop integration. We compared the difference between the average errors—with respect to the true trajectories—of simulated (Sim) trajectories vs. surrogate (Surr) trajectories formed using uniformly randomly sampled derivatives drawn from the overall distribution of observed derivatives. The relatively thinner red lines indicate the upper and lower limits of the 95% confidence interval of the distribution of simulation errors. The thick middle red line is indicative of the mean simulation error. The light gray lines depict the individual simulation error trajectories.

Neurally decoded valence and arousal processing temporal derivatives were successfully used to simulate affect processing trajectories according to closed-loop numerical integration. Numerical integration of the true temporal sequence of neurally decoded derivatives for each participant demonstrated that these decodings exhibit significantly less (*p* < 0.05, *t*-test, h_0_: μ_1_-μ_2_ = 0) group-level simulation error than integration of decoded derivatives sampled uniformly randomly from the same sequence, depicted in Figure 3. These errors were averaged over 30 iterations of the simulation. Moreover, simulations of valence and arousal processing were significant for up to four steps of closed-loop simulation (Δt = 2.0 s) for both valence and arousal, respectively. As an additional validation of these simulations, we modeled within-subject root mean squared error (RMSE) of the closed-loop first derivatives as a function of the number of simulation steps (from 1 to 6 steps) according to iteratively reweighted least-squares regression (Holland and Welsch, 1977). We then calculated the group-level mean RMSE of these growth models, which we found to be 0.37 Likert-scale units per simulation step (95% CI [0.35–0.40], *p* < 0.001, *t*-test, h_0_: β = 0) for valence and 0.37 Likert-scale units per simulation step (95% CI [0.35–0.39], *p* < 0.001, *t*-test, h_0_: β = 0) for arousal.

### Neural Encodings of Resting State Affect Processing Dynamics

**Figure 4:**
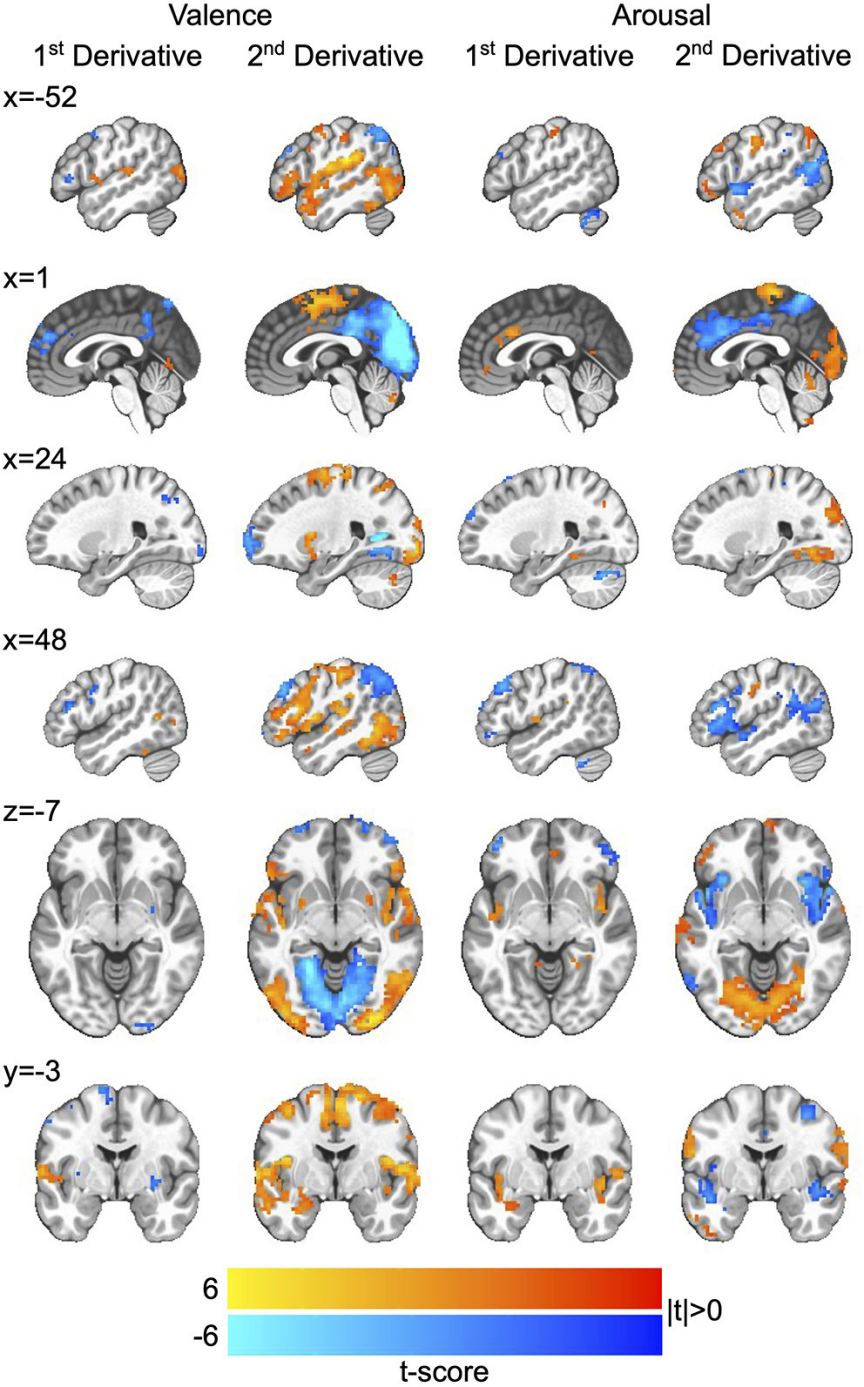
Neurocircuitry of affect processing dynamics. Group-level neural encodings of the first and second temporal derivatives of resting state valence and arousal processing. T-scores are presented only for those voxels in which encoding parameters survived global permutation testing (*p* < 0.05, *N* = 500 random permutations) as well as cluster-size thresholding ≥20 voxels (measured as face-wise nearest neighbors). Slices are presented in MNI coordinate space and neurological convention (image left equals participant left). Voxel intensities are depicted as colors having a maximum absolute intensity of |t| = 6.0, i.e., color saturates for absolute t-scores above the maximum intensity.

We then applied the Haufe-transform (Haufe et al., 2014) to form neural encoding representations of the neural decoding models by which we simulated affect processing. The resultant group-level encodings for both first and second derivatives, respectively, of the processing of affective valence and arousal are presented in Figure 4. Due to the profuse activations throughout the brain across the encoding, we summarize key observations here and refer those interested in detailed analysis to the raw encoding maps, which are publicly available (see Data Availability Statement).

#### Affective Arousal Processing Dynamics

The processing of affective arousal dynamics is distributed across broad networks of regions that exhibit both shared and distinct dynamic roles. The first temporal derivative of arousal processing is positively encoded by a network comprising the anterior cingulate cortex (aCC), bi-lateral insula, bilateral lingual gyrus, right superior occipital gyrus, left precuneus, and left superior frontal gyrus as well as a smaller right-lateralized network comprising negative encodings of the middle frontal gyrus, superior parietal lobule, and middle orbital gyrus.

The second temporal derivative of arousal processing is highly lateralized by positive vs. negative encoding. Positive encoding derives from a left-lateralized network composed of the ventral lateral prefrontal cortex (vlPFC), middle frontal gyrus, inferior frontal gyrus, and angular gyrus as well as the supplementary motor area and bi-lateral somatosensory cortex. Negative encodings derive from a right-lateralized network composed of dorsolateral prefrontal cortex (dlPFC), vlPFC, and the aCC and a bi-lateral posterior middle temporal gyrus (pmTG).

#### Affective Valence Processing Dynamics

Similar to that of arousal, the processing of affective valence dynamics is widely distributed throughout the brain. The first temporal derivative of valence processing is positively encoded by a network comprising the left superior temporal gyrus and left medial temporal pole as well as right posterior middle temporal gyrus and right inferior parietal lobule (IPL); it is negative encoded by the superior medial gyrus, bi-lateral inferior frontal gyrus, left middle frontal gyrus, bi-lateral putamen, right precentral gyrus, and precuneus.

The second temporal derivative of valence processing is represented by an extensively distributed network comprising positive encodings in the right dlPFC, bi-lateral putamen, left amygdala, left superior temporal gyrus, right somatosensory cortex, and bi-lateral pmTG as well as negative encodings in bi-lateral middle frontal gyrus, posterior cingulate cortex, precuneus, bi-lateral IPL, and the primary visual cortex.

## Discussion

In this study, we applied neural decoding models of affect processing in order to characterize low-dimensional (valence and arousal) moment-to-moment affect processing dynamics (operationalized as temporal derivatives) that arise within the untasked human brain. We then constructed and validated neural decoding models of these derivatives, thus defining the neurally constrained ordinary differential equations (ODEs) that drive resting state affect processing dynamics. From these models, we made two important contributions to the affective neuroscience and neuroimaging literature. First, we demonstrated that the neurally-derived ODEs accurately simulated low-dimensional affect processing dynamics, thereby computationally validating our proposed novel framework for analyzing the dynamics of resting state fMRI data. Critically, our proposed approach used out-of-sample ensemble averaging to decode affect processing in hold-out subjects. Thus, our approach can potentially be scaled to resting state fMRI datasets for which no affect induction task-related fMRI data is available, and, therefore, no neural decoding models of affect processing could be constructed. Second, our approach allowed for the neurally-derived ODEs to be transformed to encoding models such that the neural correlates of affect processing dynamics could be identified. To our knowledge, this is the first time the neurocircuits driving these dynamics have been observed. Overall, we found that affect processing dynamics are driven by widely distributed networks that differ between the valence and arousal dimensions of affect processing. We also found that several regions perform encoding roles across affective properties as well as orders of temporal dynamics.

Overall, the identified encoding regions of affect processing dynamics overlap with well-established regions associated with the encoding of task-related affective reactivity and regulation, which include the sensory motor area (SMA), pre-SMA, dACC, dlPFC, vlPFC, amygdala, insula, and ventral medial PFC (Lindquist et al., 2012; Etkin et al., 2015). Our work adds additional context to these findings, providing a map of how activation in these regions may signal moment-to-moment changes in overall affective experience. Perhaps more intriguing is that we found the second derivative of affect processing dynamics to be more robustly encoded than the first derivative. Specifically, there is a greater total number of significant voxels present in the group-level neural encoding representations of the second derivative as visually depicted in both Figure 4 and the raw encoding maps. This finding was true across the independent dimensions of affective valence and arousal. At present, we hypothesize that this finding suggests one of two possibilities. First, this difference may suggest that one or more regions may encode both positive and negative instantaneous change. As specified, our decoding approach would not detect regions performing this processing role. Second, instantaneous affect processing change (first derivative) may require relatively less information processing relative to the second derivative. For example, second derivative dynamics are more indicative of regulation processes rather than a measure of system state, which may draw upon multiple levels of cognition such as goal formation, rule-set maintenance, and planning. Additional experimentation may be necessary to disambiguate the potential presence of these complex cognitions from the overall encoding of the second derivative that we depict here.

### Limitations

As with all machine learning studies, our decoding model predictions (on which we built the primary findings of this work) relied extensively on the quality of the fMRI-derived features and their labels. We have previously reported on the limitations of exploiting IAPS normative scores as affective labels for training predictive models (Bush et al., 2018a,b; Wilson et al., 2020). We have also previously reported on the limitations of neural decoding of affect processing in populations, such as the one reported here, which diverge from the population on which the IAPS images' normative scores were collected (Wilson et al., 2020). Both age (Mather and Knight, 2005; Grühn and Scheibe, 2008; Charles and Piazza, 2009) and sex (Sabatinelli et al., 2004; McRae et al., 2008; Domes et al., 2009) are known to impact participants' affective experiences, which may bias the underlying decoding models, and, therefore, alter our group-level encodings of affect processing dynamics, which are derived from these base measures.

### Broader Contributions

The analysis framework proposed in this work is a specific case of a more general neuroimaging framework that could be extended to explore the dynamics of other cognitive processes beyond that of affect. This framework proposes the use of neural decoding models, trained out-of-sample, to project resting state fMRI data into low-dimensional, cognition-specific state spaces within which neural decoding models of process dynamics may be constructed, validated (via simulation), and subsequently encoded in order to elucidate the underlying neural mechanisms. This framework may also, given representative sets of neural decoding models, provide a way of understanding group-level differences in cognitive processing dynamics, e.g., healthy vs. psychopathological populations, and may lead to novel biomarkers for characterizing psychopathologies. The framework could also be extended to pediatric populations if the challenge of significant motion artifact in these populations is overcome to therefore permit the building of high-quality decoding models. We recognize the potential impact of this work in applications to the large-scale ABCD study dataset (Casey et al., 2018).

## Data Availability Statement

The authors have made the source code used to conduct this analysis publicly available: https://github.com/kabush/IN2. All reported functional neuroanatomical activation maps are publicly available via the study's Open Science Framework repository: https://osf.io/4gqtu. De-identified CTM study data is also publicly available as a Brain Imaging Data Structure (BIDS) formatted dataset: 10.18112/openneuro.ds003831.v1.0.0. Note, this dataset does not include the (*n* = 19) INCA study subjects who did not consent to public release of their de-identified data. For replication purposes only, de-identified raw data from the INCA study may be made privately available upon request.

## Ethics Statement

The studies involving human participants were reviewed and approved by the UAMS Institutional Review Board. The patients/participants provided their written informed consent to participate in this study.

## Author Contributions

KF and KB: conception, design, implementation, testing, analysis, interpretation of results, manuscript preparation, and revisions. Both authors contributed to the article and approved the submitted version.

## Funding

This study was funded by National Science Foundation grant BCS-1735820 (KB). Additional personnel support was provided by National Institute on Drug Abuse grant 1T32DA022981 (KF). Subject recruitment for the project was supported by the UAMS Translational Research Institute (TRI) through the National Center for Advancing Translational Sciences (1U54TR001629-01A1).

## Conflict of Interest

The authors declare that the research was conducted in the absence of any commercial or financial relationships that could be construed as a potential conflict of interest.

## Publisher's Note

All claims expressed in this article are solely those of the authors and do not necessarily represent those of their affiliated organizations, or those of the publisher, the editors and the reviewers. Any product that may be evaluated in this article, or claim that may be made by its manufacturer, is not guaranteed or endorsed by the publisher.

## References

[B1] American Psychiatric Association (1994). Diagnostic and Statistical Manual of Mental Disorders, Fourth Edition (DSM-IV). Washington, DC: American Psychiatric Association.

[B2] Berking M. Wupperman P. (2012). Emotion regulation and mental health: recent findings, current challenges, and future directions. Curr. Opin. Psychiatry. 25, 128–134. 10.1097/YCO.0b013e3283503669 22262030

[B3] Brown S. A. Brumback T. Tomlinson K. Cummins K. Thompson W. K. Nagel B. J.. (2015). The national consortium on alcohol and neuro-development in adolescence (NCANDA): a multisite study of adolescent development and substance use. J. Stud. Alcohol Drugs 76, 895–908. 10.15288/jsad.2015.76.895 26562597 PMC4712659

[B4] Bullmore E. Sporns O. (2009). Complex brain networks: graph theoretical analysis of structural and functional systems. Nat. Rev. Neurosci. 10, 186–198. 10.1038/nrn2575 19190637

[B5] Bush K. A. Gardner J. Privratsky A. Chung M.-H. James G. A.. (2018b). Brain states that encode perceived emotion are reproducible but their classification accuracy is stimulus-dependent. Front Hum Neurosci. 12, 262. 10.3389/fnhum.2018.00262 30013469 PMC6036171

[B6] Bush K. A. James G. A. Privratsky A. A. Fialkowski K. P. Kilts C. D. (2020). An action-value model explains the role of the dorsal anterior cingulate cortex in performance monitoring during affect regulation. bioRxiv. 23. 10.1101/2020.09.08.283671 PMC9426889 36040991

[B7] Bush K. A. Privratsky A. Gardner J. Zielinski M. J. Kilts C. D. (2018a). Common functional brain states encode both perceived emotion and the psychophysiological response to affective stimuli. Sci. Rep. 8, 15444. 10.1038/s41598-018-33621-6 30337576 PMC6194055

[B8] Bush K. A. Privratsky A. A. Kilts C. D. (2018c). “Predicting affective cognitions in the resting adult brain,” in Proceedings of the Conference on Cognitive Computational Neuroscience (Philadelphia, PA).

[B9] Buxton R. B. Uludag K. Dubowitz D. J. Liu T. T. (2004). Modeling the hemodynamic response to brain activation. Neuroimage 23, S220–S233. 10.1016/j.neuroimage.2004.07.013 15501093

[B10] Calhoun V. D. Miller R. Pearlson G. Adali T. (2014). The chronnectome: time-varying connectivity networks as the next frontier in fMRI data discovery. Neuron 84, 262–274. 10.1016/j.neuron.2014.10.015 25374354 PMC4372723

[B11] Casey B. J. Cannonier T. Conley M. I. Cohen A. O. Barch D. M. Heitzeg M. M. (2018). The adolescent brain cognitive development (ABCD) study: imaging acquisition across 21 sites. Dev. Cogn. Neurosci. 32, 43–54. 10.1016/j.dcn.2018.03.001 29567376 PMC5999559

[B12] Charles S. T. Piazza J. R. (2009). Age differences in affective well-being: context matters: aging and effect. Soc. Pers. Psychol. Compass 3, 711–724. 10.1111/j.1751-9004.2009.00202.x

[B13] Cisler J. M. Elton A. Kennedy A. P. Young J. Smitherman S. Andrew James G. (2013). Altered functional connectivity of the insular cortex across prefrontal networks in cocaine addiction. Psychiatry Res. Neuroimaging 213, 39–46. 10.1016/j.pscychresns.2013.02.007 23684980 PMC3708551

[B14] Cox R. W. (1996). AFNI: software for analysis and visualization of functional magnetic resonance neuroimages. Comput. Biomed. Res. 29, 162–173. 10.1006/cbmr.1996.0014 8812068

[B15] Cox R. W. (2002). “Outlier detection in fMRI time series,” in Proceedings of International Society for Magnetic Resonance in Medicine (Concord, CA).

[B16] Cunningham W. A. Dunfield K. A. Stillman P. E. (2013). Emotional states from affective dynamics. Emotion Rev. 5, 344–355. 10.1177/1754073913489749

[B17] Dietterich T. G. (2000). “Ensemble methods in machine learning,” in Multiple Classifier Systems, Lecture Notes in Computer Science; vol. 1857, eds G. Goos, J. Hartmanis, and J. van Leeuwen (Berlin; Heidelberg: Springer Berlin Heidelberg).

[B18] Domes G. Schulze L. Böttger M. Grossmann A. Hauenstein K. Wirtz P. H. (2009). The neural correlates of sex differences in emotional reactivity and emotion regulation. Hum. Brain Mapp. 31, 758–769. 10.1002/hbm.20903 19957268 PMC6871188

[B19] Dosenbach N. U. F. Nardos B. Cohen A. L. Fair D. A. Power J. D. Church J. A. (2010). Prediction of individual brain maturity using fMRI. Science 329, 1358–1361. 10.1126/science.1194144 20829489 PMC3135376

[B20] Etkin A. Büchel C. Gross J. J. (2015). The neural bases of emotion regulation. Nat. Rev. Neurosci. 16, 693–700. 10.1038/nrn4044 26481098

[B21] Glasser M. F. Coalson T. S. Robinson E. C. Hacker C. D. Harwell J. Yacoub E. (2016). A multi-modal parcellation of human cerebral cortex. Nature 536, 171–178. 10.1038/nature18933 27437579 PMC4990127

[B22] Gonzalez-Castillo J. Caballero-Gaudes C. Topolski N. Handwerker D. A. Pereira F. Bandettini P. A. (2019). Imaging the spontaneous flow of thought: distinct periods of cognition contribute to dynamic functional connectivity during rest. NeuroImage 202, 116129. 10.1016/j.neuroimage.2019.116129 31461679 PMC6819261

[B23] Gonzalez-Castillo J. Hoy C. W. Handwerker D. A. Robinson M. E. Buchanan L. C. Saad Z. S. (2015). Tracking ongoing cognition in individuals using brief, whole-brain functional connectivity patterns. Proc. Natl. Acad. Sci. U.S.A. 112, 8762–8767. 10.1073/pnas.1501242112 26124112 PMC4507216

[B24] Gordon E. M. Laumann T. O. Gilmore A. W. Newbold D. J. Greene D. J. Berg J. J. (2017). Precision functional mapping of individual human brains. Neuron 95, 791–807.e7. 10.1016/j.neuron.2017.07.011 28757305 PMC5576360

[B25] Greicius M. D. Flores B. H. Menon V. Glover G. H. Solvason H. B. Kenna H. (2007). Resting-state functional connectivity in major depression: abnormally increased contributions from subgenual cingulate cortex and thalamus. Biol. Psychiatry 62, 429–437. 10.1016/j.biopsych.2006.09.020 17210143 PMC2001244

[B26] Greicius M. D. Srivastava G. Reiss A. L. Menon V. (2004). Default-mode network activity distinguishes Alzheimer's disease from healthy aging: evidence from functional MRI. Proc. Natl. Acad. Sci. U.S.A. 101, 4637–4642. 10.1073/pnas.0308627101 15070770 PMC384799

[B27] Grühn D. Scheibe S. (2008). Age-related differences in valence and arousal ratings of pictures from the International Affective Picture System (IAPS): do ratings become more extreme with age? Behav. Res.Methods 40, 512–521. 10.3758/BRM.40.2.512 18522062

[B28] Gu S. Satterthwaite T. D. Medaglia J. D. Yang M. Gur R. E. Gur R. C. (2015). Emergence of system roles in normative neurodevelopment. Proc. Natl. Acad. Sci. U.S.A. 112, 13681–13686. 10.1073/pnas.1502829112 26483477 PMC4640772

[B29] Haufe S. Meinecke F. Görgen K. Dähne S. Haynes J-.D.. (2014). On the interpretation of weight vectors of linear models in multivariate neuroimaging. Neuroimage 87, 96–110. 10.1016/j.neuroimage.2013.10.067 24239590

[B30] Holland P. W. Welsch R. E. (1977). Robust regression using iteratively reweighted least-squares. Commun. Stat. 6, 813–827. 10.1080/03610927708827533

[B31] Jenkinson M. Beckmann C. F. Behrens T. E. J. Woolrich M. W. Smith S. M. (2012). FSL. Neuroimage 62, 782–790. 10.1016/j.neuroimage.2011.09.015 21979382

[B32] Kecman V. Huang T.-M. Vogt M. (2005). “Iterative single data algorithm for training kernel machines from huge data sets: theory and performance,” in Support Vector Machines: Theory and Applications Studies in Fuzziness and Soft Computing (Berlin: Springer), p. 255–574.

[B33] Killingsworth M. A. Gilbert D. T. (2010). A wandering mind is an unhappy mind. Science 330, 932–932. 10.1126/science.1192439 21071660

[B34] Kragel P. A. Hariri A. R. LaBar K. S. (2021). The temporal dynamics of spontaneous emotional brain states and their implications for mental health. J. Cogn. Neurosci. 1–14. 10.1162/jocn_a_01787 34705046 PMC9026845

[B35] Kragel P. A. Knodt A. R. Hariri A. R. LaBar K. S. (2016). Decoding spontaneous emotional states in the human brain. PLoS Biol. 14, e2000106. 10.1371/journal.pbio.2000106 27627738 PMC5023171

[B36] Lang P. J. Bradley M. M. Cuthbert B. N. (2008). International Affective Picture System (IAPS): Affective Ratings of Pictures and Instruction Manual. Gainesville, FL: University of Florida; Report No.: Technical Report A-8.

[B37] Lindquist K. A. Wager T. D. Kober H. Bliss-Moreau E. Barrett L. F. (2012). The brain basis of emotion: a meta-analytic review. Behav. Brain Sci. 35, 121–143. 10.1017/S0140525X11000446 22617651 PMC4329228

[B38] Liu X. Duyn J. H. (2013). Time-varying functional network information extracted from brief instances of spontaneous brain activity. Proc. Natl. Acad. Sci. U.S.A. 110, 4392–4397. 10.1073/pnas.1216856110 23440216 PMC3600481

[B39] Mather M. Knight M. (2005). Goal-directed memory: the role of cognitive control in older adults' emotional memory. Psychol. Aging 20, 554–570. 10.1037/0882-7974.20.4.554 16420131

[B40] McRae K. Ochsner K. N. Mauss I. B. Gabrieli J. J. D. Gross J. J. (2008). Gender differences in emotion regulation: an fMRI study of cognitive reappraisal. Group Process. Intergroup Relat. 11, 143–162. 10.1177/1368430207088035 29743808 PMC5937254

[B41] Mitchell T. M. Shinkareva S. V. Carlson A. Chang K-.M. Malave V. L.. (2008). Predicting human brain activity associated with the meanings of nouns. Science. 320, 1191–1195. 10.1126/science.1152876 18511683

[B42] Parzen E. (2015). Stochastic Processes. Mineola, NY: Dover Publications.

[B43] Power J. D. Barnes K. A. Snyder A. Z. Schlaggar B. L. Petersen S. E. (2012). Spurious but systematic correlations in functional connectivity MRI networks arise from subject motion. Neuroimage 59, 2142–2154. 10.1016/j.neuroimage.2011.10.018 22019881 PMC3254728

[B44] Power J. D. Schlaggar B. L. Petersen S. E. (2015). Recent progress and outstanding issues in motion correction in resting state fMRI. Neuroimage 105, 536–551. 10.1016/j.neuroimage.2014.10.044 25462692 PMC4262543

[B45] Privault N. (2013). Understanding Markov Chains: Examples and Applications. Berlin: Springer

[B46] Raichle M. E. MacLeod A. M. Snyder A. Z. Powers W. J. Gusnard D. A. Shulman G. L. (2001). A default mode of brain function. Proc. Natl. Acad. Sci. U.S.A. 98, 676–682. 10.1073/pnas.98.2.676 11209064 PMC14647

[B47] Rissman J. Gazzaley A. D'Esposito M. (2004). Measuring functional connectivity during distinct stages of a cognitive task. Neuroimage 23, 752–763. 10.1016/j.neuroimage.2004.06.035 15488425

[B48] Sabatinelli D. Flaisch T. Bradley M. M. Fitzsimmons J. R. Lang P. J. (2004). Affective picture perception: gender differences in visual cortex? Neuroreport 15, 1109–1112. 10.1097/00001756-200405190-00005 15129155

[B49] Smith S. M. Beckmann C. F. Andersson J. Auerbach E. J. Bijsterbosch J. Douaud G. (2013). Resting-state fMRI in the human connectome project. Neuroimage 80, 144–168. 10.1016/j.neuroimage.2013.05.039 23702415 PMC3720828

[B50] Thomas J. W. (1995). “Numerical partial differential equations: finite difference methods,” in Texts in Applied Mathematics, Vol. 22 (New York, NY: Springer-Verlag).26034665

[B51] Wilson K. A. James G. A. Kilts C. D. Bush K. A. (2020). Combining Physiological and neuroimaging measures to predict affect processing induced by affectively valent image stimuli. Sci. Rep. 10, 9298. 10.1038/s41598-020-66109-3 32518277 PMC7283349

[B52] Zelazo P. D. Cunningham W. A. (2007). “Executive function: mechanisms underlying emotion regulation” in Handbook of Emotion Regulation (New York, NY: The Guilford Press), 135–58.

