# Supplementary material for: Identifying the Neural Correlates of Resting State Affect Processing Dynamics
Source: Front Neuroimaging. 2022 Apr 21;1:825105. doi: 10.3389/fnimg.2022.825105 (PMC10406310; doi:10.3389/fnimg.2022.825105)
Supplement: Supplementary file 1 [file Table_1.DOCX]

Supplementary Material

# Supplementary Figures


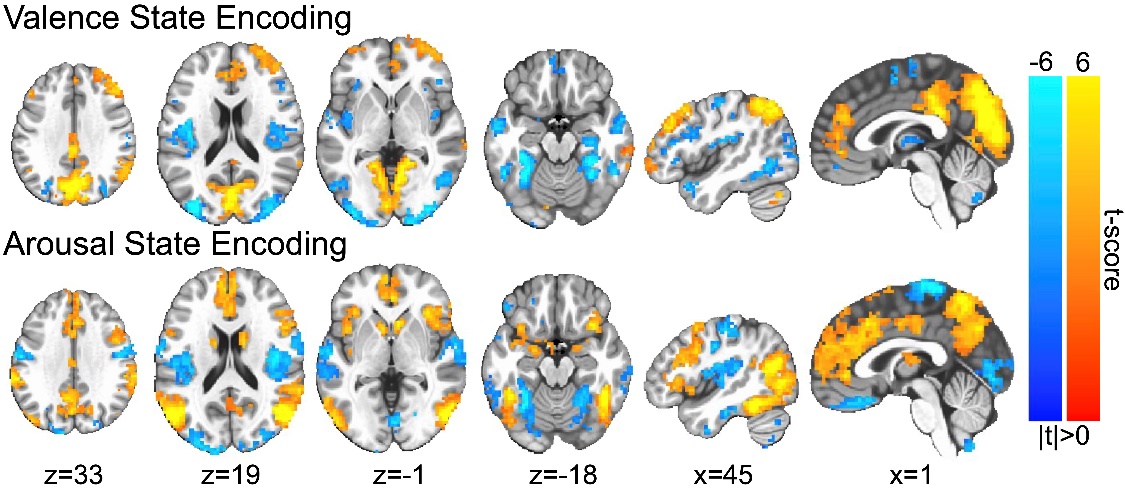


**Supplementary Figure 1.** Neural encodings of affect processing. Color gradations indicate the group-level t-scores of the encoding parameters (red indicating positive valence or high arousal, blue indicating negative valence or low arousal). T-scores are presented only for those voxels in which encoding parameters survived global permutation testing (p<0.01, uncorrected, N=1000 random permutations). Image slices are presented in MNI coordinate space and neurological convention. Maximum voxel intensity is |t|=6.0, i.e., color saturates for t-scores with absolute values falling above this value. Note, this figure is reproduced from Bush et al. (2020)[1].

# Supplementary References

1. Bush KA, James GA, Privratsky AA, Fialkowski KP, Kilts CD. Action-value processing underlies the role of the dorsal anterior cingulate cortex in performance monitoring during self-regulation of affect. bioRxiv. 2020;23. DOI: 10.1101/2020.09.08.283671
